# Rapamycin-Induced Apoptosis in HGF-Stimulated Lens Epithelial Cells by *AKT*/*mTOR*, *ERK* and *JAK2*/*STAT3* Pathways

**DOI:** 10.3390/ijms150813833

**Published:** 2014-08-11

**Authors:** Fang Tian, Lijie Dong, Yu Zhou, Yan Shao, Wenbo Li, Hong Zhang, Fei Wang

**Affiliations:** 1 Tianjin Medical University Eye Hospital& Eye Institute, No. 251, Fu Kang Road, Nan Kai District, Tianjin 300384, China; E-Mails: tianfang1216@126.com (F.T.); snail.mutou@163.com (Y.Z.); 13752613234@163.com (Y.S.); wenboli0412@126.com (W.L.); tmuechong@sina.com (H.Z.); 2 Institute of Innovative Medicine and Food Safety, Jiangsu Berkgen Biopharmaceutical Inc., Ltd., No. 20, Jian Hua Road, Hang Jiang District, Yangzhou 225128, China

**Keywords:** rapamycin, apoptosis, *AKT*/*mTOR*, *ERK*, *JAK2*/*STAT3*

## Abstract

Hepatocyte growth factor (HGF) induced the proliferation of lens epithelial cells (LECs) and may be a major cause of posterior capsule opacification (PCO), which is the most frequent postoperative complication of cataract surgery. To date, several agents that can block LECs proliferation have been studied, but none have been used in clinic. Recently, accumulating evidence has suggested rapamycin, the inhibitor of *mTOR* (mammalian target of Rapamycin), was associated with the induction of apoptosis in LECs. The purpose of our study was to investigate the potential effects of rapamycin on HGF-induced LECs and the underlying mechanisms by which rapamycin exerted its actions. Using cell proliferation, cell viability and flow cytometric apoptosis assays, we found that rapamycin potently not only suppressed proliferation but also induced the apoptosis of LECs in a dose-dependent manner under HGF administration. Further investigation of the underlying mechanism using siRNA transfection revealed that rapamycin could promote apoptosis of LECs via inhibiting HGF-induced phosphorylation of *AKT*/*mTOR*, *ERK* and *JAK2*/*STAT3* signaling molecules. Moreover, the forced expression of *AKT*, *ERK* and *STAT3* could induce a significant suppression of apoptosis in these cells after treatment of rapamycin. Together, these findings suggested that rapamycin-induced apoptosis in HGF-stimulated LECs is accompanied by inhibition of *AKT*/*mTOR*, *ERK* and *JAK2*/*STAT3* pathways, which supports its use to inhibit PCO in preclinical studies and provides theoretical foundation for future possible practice.

## 1. Introduction

The most frequent postoperative complication of cataract surgery that will result in visual loss is posterior capsule opacification (PCO), which is mainly caused by the proliferation and migration of postoperative remnants of lens epithelial cells (LECs) in the posterior lens capsule [1,2,3,4,5]. Nd: YAG laser posterior capsulotomy is always required to restore vision [6]. However, it may increase the risk of retinal detachment, hemorrhage and lens injury, *etc.* [7]. Moreover, in terms of children patients, prone to PCO, laser therapy could not be performed successfully because of their poor cooperation. Therefore, noninvasive means of preventing PCO while maintaining the integrity of the posterior capsule are being investigated. To date, several drugs that can block LECs proliferation have already been studied, but none of them has been used in practice.

The cytokines that are produced by the LECs themselves are believed to be important in the development of PCO [8,9]. Earlier studies analyzed hepatocyte growth factor (HGF) and found it to be highly expressed in human capsular bag at all stages of cultures in a protein-free medium, and to induce proliferation of LECs in human LEC lines and in capsular bag cultures, and suggesting HGF may play an important role in the development of PCO [3,10,11]. Although LECs proliferation in response to HGF has been well documented [3,10], the molecular mechanisms responsible for the termination of HGF signaling are still unknown.

Rapamycin, also known as Sirolimus, is a natural product isolated from *Streptomyces hygroscopicus* [12,13], a potent immunosuppressive drug which has been found to be able to inhibit proliferation of rabbit LECs *in vitro* [14] and promote the apoptosis in human LECs (HLECs) [15,16], and is currently used as an anti-tumor agent and an immunomodulatory medication, characterized with low toxicity and high efficiency [12,17,18,19,20,21,22,23]. Thus, rapamycin may be a potential inhibitor of PCO.

Therefore, the following study was performed to investigate the inhibition of HLECs proliferation and/or induction of its apoptosis promoted by rapamycin in the presence of HGF. This is the first study to identify the miscellaneous pathways of rapamycin acting on HLECs treated by HGF, which will indeed contribute to develop a new strategy to prevent PCO.

## 2. Results and Discussion

### 2.1. Impact of Rapamycin on the Proliferation in HGF-Treated Lens Epithelial Cells (LECs)

**Figure 1:**
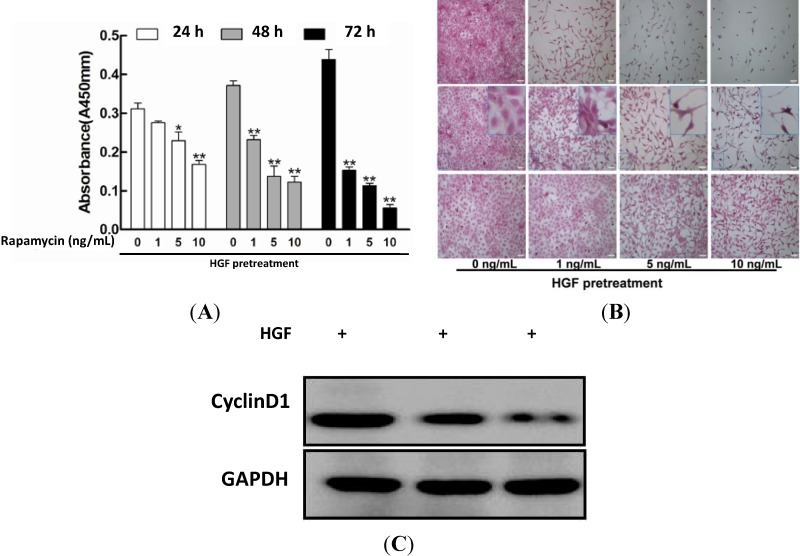
Rapamycin inhibited the proliferation of hepatocyte growth factor (HGF)-induced lens epithelial cells (LECs) in dose-and time-dependent manners. After pretreatment with HGF (10 ng/mL) for 12h, LECs were dealt with increasing doses of rapamycin(0, 1,5 and 10 ng/mL) for different time points (24, 48 and 72 h). (**A**) The proliferation assay (ELISA BrdU kit), Each bar represents the mean ± SD of the group, * *p* < 0.05, ** *p* < 0.01, compared with control cells(rapamycin, 0 ng/mL).(*F*_24 h_ *=* 18.986, *F*_48 h_ *=* 45.105, *F*_72 h_ = 141.161, scheffe’s multiple range test); (**B**) H&E staining, after rapamycin stimulation, HGF-pretreated LECs showed impaired proliferative abilities in dose and time dependent manners(scale bar:50μm); and (**C**) Western blotting, rapamycin treatment decreased the expression of cyclin D1 after HGF pretreatment.

The proliferation assay was performed to determine the proliferation rate respectively by Cell Proliferation ELISA BrdU kit (Roche, 11647229001, Mannheim, Germany). As shown in Figure 1A, compared to the control group, at 24 h rapamycin (5 or 10 ng/mL) could significantly repress LECs proliferation (* *p* < 0.05) and at 48 h the proliferation rate was down-regulated by rapamycin (1 ng/mL). These data suggested that rapamycin inhibited HGF-induced proliferation of LECs in a dose and time dependent manner. Meanwhile, we used H&E staining to detect the morphology of LECs respectively with the rapamycin treatment. As shown in Figure 1B, untreated cells were large and full, cytoplasmic staining was uniform, and the nucleus were round as well. After rapamycin stimulation, there were an obvious declination of the proliferative capacity in a dose and time dependent manner and the cells showed characteristic apoptotic changes, such as smaller, chromatin margination, nuclear hyperchromatism, pyknosis, *etc.* Following an increasing in working concentration and incubation time of rapamycin, the changes in cell morphology turned much more obvious and the number of LECs appeared to be even less. After treating with rapamycin for different time points as indicated, we detected the expression of cyclin D1 in LECs. Consistent with the reduction of proliferation in LECs, the level of cyclin D1 was decreased accordingly (Figure 1C).

The lens epithelium proliferation and its epithelium-mensenchymal transition (EMT) is involved together in PCO. Rapamycin, an *mTOR* inhibitor, can strongly inhibit proliferation, fibroblast growth factor induced migration, and extracellular matrix fibronectin formation in rabbit LECs [14]. Moreover, it has been verified that *mTOR* is activated during the TGF-β2-induced EMT in a time-dependent manner.Therefore, not only from the view of proliferation, but from EMT as well, rapamycin has the ability to control PCO [24]. An intraocular lens coating with polylactideglycoli acid and rapamycin effectively prevented formation and development of PCO for a relatively long duration in rabbit eyes [15,16]. The expression of pro-apoptotic protein Bax has often been associated with the increased apoptosis, while the anti-apoptotic protein Bcl-2 has been related with the inhibition of apoptosis in target cells [25,26,27]. Rapamycin could suppress the expression of Bcl-2 protein, but promote the expression of Bax protein which suggested that rapamycin could inhibit proliferation of LECs probably not only by blocking the progress of cell cycle, but also by promoting the induction of apoptosis. In the past few years, a new strategy was proposed to remove the residual LECs by inducing apoptosis [16]. Previous studies have shown that over expression of Bax or procaspase-3 is capable of inducing therapeutic programmed cell death of residual LECs *in vitro* and *in vivo* and preventing PCO in a rabbit model [28,29]. Recent studies have concentrated on the cytokines involved in the development of PCO that were produced by the LECs themselves and believed to play an important role [3,30,31,32,33,34]. In fact, HGF was up-regulated in the remaining LECs after cataract surgery by unknown mechanisms and it has been found to enhance protein synthesis, proliferation, and migratory rates of human lens cell line (FHL124) by activation of c-met receptor [10]. Thus HGF may contribute to the development of PCO after cataract surgery; therefore, we used HGF to treat LECs cells to simulate the post cataract surgery environments.

### 2.2. Effect of Rapamycin on the Apoptosis in HGF-Treated LECs

**Figure 2:**
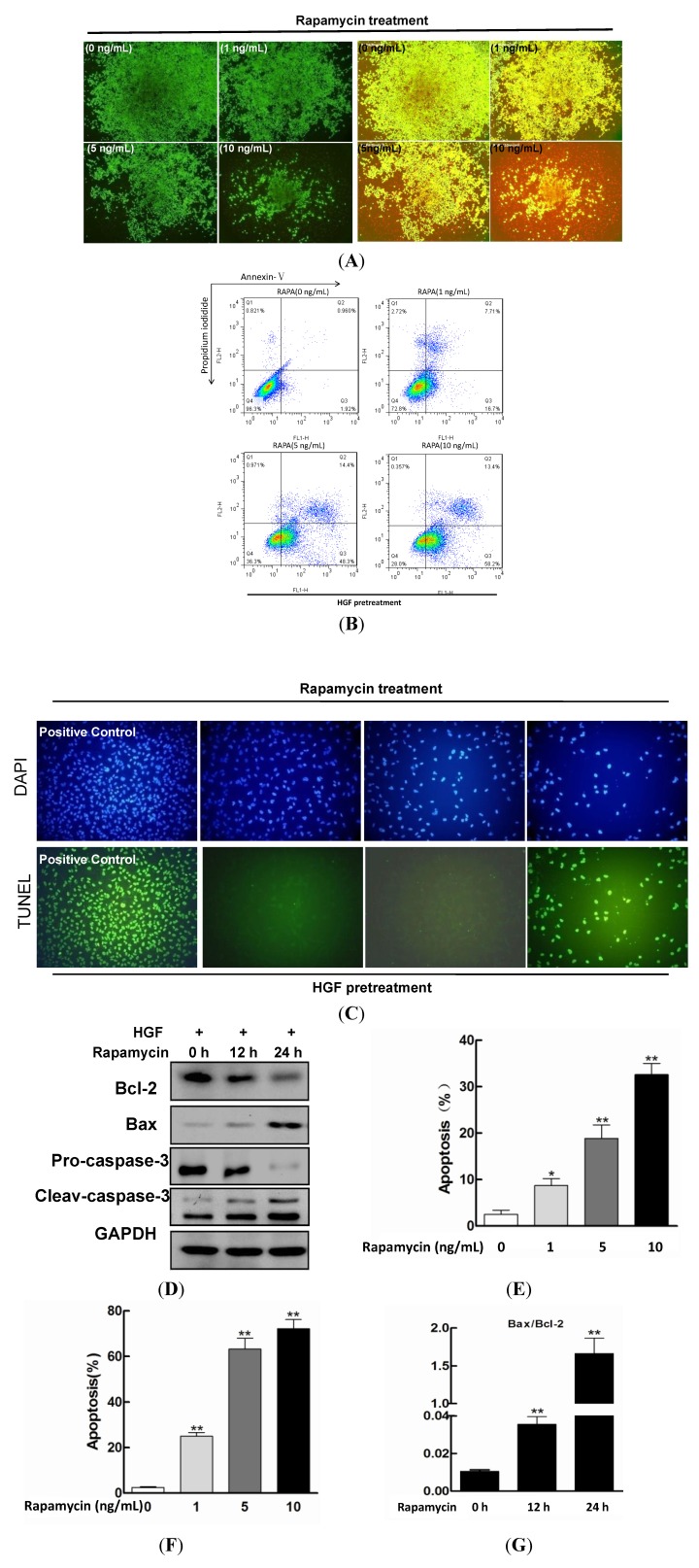
Rapamycin treatment induced apoptosis in HGF-pretreated LECs cells. HGF (10 ng/mL)-pretreated LECs cells were managed with rapamycin (0, 1, 5 and 10 ng/mL) for 48 h. (**A**) LIVE & DEAD Viability/Cytotoxicity assay; (**B**) AnnexinV and PI staining were used to quantify the percentage of apoptotic cells; (**C**) Tunnel assay. The increased number of TUNEL-positive cells along with the increasing concentration of rapamycin treatment clearly demonstrated that the rapamycin could induce apoptosis of LECs cells *in vitro*; (**D**) Western Blotting analysis. HGF (10 ng/mL)-pretreated LECs were loaded with rapamycin (5 ng/mL) for 0, 12 or 24 h. The expression of apoptosis-related proteins, including Bcl-2, Bax, pro-caspase-3 and cleave-caspase-3 were shown with GAPDH as a control; (**E**) Quantitative analysis of the apoptosis rate was shown (*F =* 40.296, scheffe’s multiple range test, * *p* < 0.05, ** *p* < 0.01); (**F**) The quantification of apoptosis induced by rapamycin was calculated (*F* *=* 102.757, scheffe’s multiple range test, ** *p* < 0.01); (**G**) Bax/Bcl-2 ratios of LECs. The densitometry value of each band was determined with Image J software. Data was presented as mean ± SD, compared with control cells (*F =* 64.570, scheffe’s multiple range test, ** *p* <0.01).

LIVE & DEAD Viability/ Cytotoxicity assay was introduced to identify the viability under rapamycin treatment with HGF stimulation. As shown in Figure 2A, living cells showed green signals while dead cells showed red signals. In negative control, there were no red signals detected under fluorescent microscopy; however, after rapamycin simulation it was remarkable that green signals decreased accompanied by a gradual increase in red signals along with the rise of rapamycin concentration. Quantification analysis confirmed that rapamycin (1 ng/mL) induced the apoptosis at 48 h (Figure 2E). These data indicated that rapamycin played a role not only in suppressing the proliferation but also inducing the death of LECs under HGF administration.

After treatment with different doses of rapamycin for 48 h, apoptosis induction was demonstrated using FCM analysis. The percentages of early and late stage apoptotic cells were shown in the lower right (Q3) and upper right (Q2) quadrant of the histograms respectively (Figure 2B). As shown in Figure 2B, there were almost normal cells and rarely visible apoptotic cells in the normal group, while in rapamycin groups, the rate of apoptotic cells gradually increased along with the augmenting concentrations of rapamycin. The total percentage of apoptotic cells (Q2 + Q3) increased from 0.96% in non-rapamycin treated LECs cells to 7.71%, 14.4% and 24.7% in rapamycin-treated cells (1, 5 and 10 ng/mL, respectively) after 48 h treatment (** *p* < 0.01) (Figure 2F). To further verify and quantify the apoptotic cells induced by rapamycin, the cells pretreated with HGF treated with or without rapamycin were applied to TUNEL assay. As shown in Figure 2C, the increased number of TUNEL-positive cells along with the increasing concentration of rapamycin treatment clearly demonstrated that the rapamycin could induce apoptosis of LECs cells *in vitro* (** *p* < 0.01).

After treating with increasing concentrations of rapamycin for 48 h, we detected the expression of Bax and Bcl-2 in LECs. Consistent with the increasing apoptosis in LECs, the level of Bax was elevated while the level of Bcl-2 had decreased (Figure 2D). The ratio of Bax/Bcl-2 protein level is the decisive factor to transmit the apoptosis signal. By comparing the intensity of their bands, we found the ratio of Bax/Bcl-2 to be augment in a dose-dependent manner (** *p* < 0.01) (Figure 2G). The downstream casepase-3 for apoptosis was subsequently activated. As is shown in Figure 2C, the expression of cyclin D1 and pro-caspase-3 decreased, while that of cleaved caspase-3 and cytochrome C was elevated.

In the present study, we demonstrated that rapamycin could potently not only inhibit proliferation but also induce apoptosis in LECs under HGF pretreatment *in vitro*. As we know, apoptosis is induced via two main routes involving either the mitochondria or the activation of death receptors. The mitochondrial pathway is activated by a wide range of signals and involves the release of proteins (including cytochrome c) from the mitochondrial membrane space [28,29,35,36]. Our results indicated that the expression of cytochrome c and the activity of cleaved caspase-3 were greatly increased after rapamycin treatment in the presence of HGF, which suggested that the apoptosis was induced by engaging mitochondrial pathway, and catalytic activation of the caspases [29]. 

### 2.3. AKT/mTOR and ERK Pathways Involvement in Apoptosis Promoted by Rapamycin in HGF-Treated LECs

**Figure 3:**
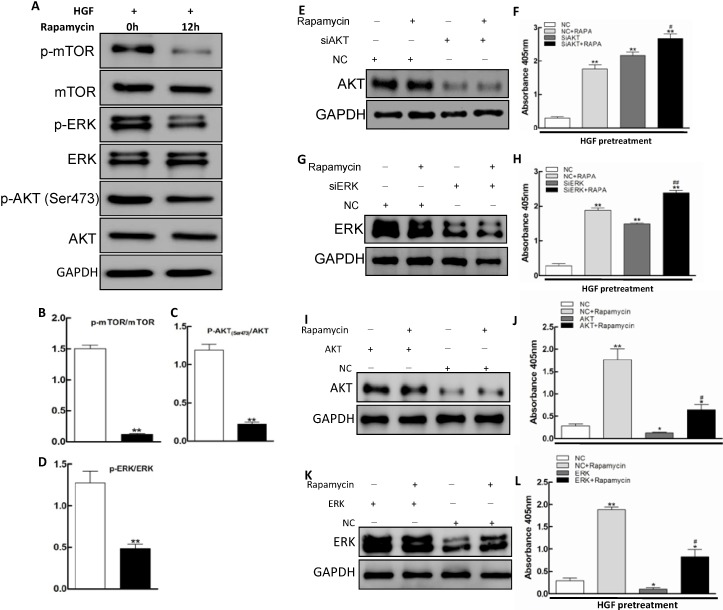
Rapamycin activated apoptosis of HGF-treated LECs by *AKT*/*mTOR* and *ERK* pathway. HGF (10 ng/mL)-pretreated LECs were stimulated with rapamycin (5 ng/mL) for 12 h or left intact. (**A**) The levels of *mTOR*, *AKT*, *ERK* and their phosphorylated forms were analyzed by western blotting with GAPDH as a control; (**B**–**D**) Quantity of the p-*ERK*/*ERK*, p-*mTOR*/*mTOR* and p-*AKT*/*AKT* ratio were determined by densitometry analysis (** *p* < 0.01, *t*-test); (**E**,**G**) *AKT* and *ERK*1/2 siRNA transfection led to a significant reduction in the *AKT* and *ERK*1/2expression; (**F**,**H**) The LECs apoptosis rates were analyzed by Cell Death Detection ELISA PLUS kit. ** *p* < 0.01,compared with the NC (negative control) LECs. ^#^ *p* < 0.05, ^##^ *p*< 0.01, compared with the LECs transfected with *AKT* or *ERK* siRNA. (*F*_F_ = 93.807, *F*_H_*=* 201.819, scheffe’s multiple range testA); (**I**,**K**) *AKT* and *ERK*1/2 forced transfection led to a significant increase in the *AKT* and *ERK*1/2expression; and (**J**,**L**) The LECs apoptosis rates were analyzed by Cell Death Detection ELISA PLUS kit* *p* <0.05, ** *p* <0.01, compared with the NC (negative control) LECs. ^#^ *p* < 0.05, compared with the LECs in forced expression of *AKT* or *ERK* (*F*_J_ = 28.208, *F*_L_= 74.277, scheffe’s multiple range test).

Rapamycin promoting apoptosis has been investigated more recently [12,37,38]. After treatment with rapamycin (5 ng/mL) in LECs cells for 48 h, the phosphorylation levels of *AKT*, *mTOR* and *ERK*1/2 were effectively suppressed (Figure 3A). The fractions of p-*AKT*/*AKT*, p-*mTOR*/*mTOR* as well as p*ERK*1/2/*ERK*1/2 were all decreased (Figure 3B–D). To investigate the role of *AKT* and *ERK* in apoptosis of LECs cells, we employed siRNA to knockdown *AKT* and *ERK*1/2 gene expression in LECs cells. As shown in Figure 3E,G, the western blot analysis indicated that transient transfection of *AKT* and *ERK*1/2 siRNA into LECs cells resulted in a significant reduction of the *AKT* and *ERK*1/2 protein levels respectively compared to the cells transfected with the control siRNA (Figure 3E,G), which was accompanied by a significant induction of apoptosis in these cells (Figure 3F,H). In addition, we also explore the function of rapamycin by forced expression of *AKT* and *ERK* gene expression in LECs cells. As shown in Figure 3I,K, the western blot analysis indicated that transient transfection of *AKT* and *ERK*1/2 into LECs cells resulted in a significant increase of the *AKT* and *ERK*1/2 protein levels respectively compared to the control group (Figure 3I,K), which was accompanied by a significant suppression of apoptosis in these cells (Figure 3J,L).

The results showed that knockdown of *AKT* or *ERK*1/2 significantly promoted apoptosis, while forced expression of *AKT* or *ERK*1/2 obviously inhibited apoptosis in HGF-treated LECs cells after rapamycin treatment, indicating that *AKT* and *ERK*1/2 played important roles in apoptosis of LECs cells with rapamycin stimulation. In addition, rapamycin enhanced the apoptosis in HGF-treated LECs cells remarkably with *AKT* or *ERK*1/2 knockdown, which suggested that inhibition of the *AKT* or *ERK* signaling pathways could enhance the pro-apoptotic effect of rapamycin in HGF treated LECs cells.

Molecular mechanisms by which rapamycin elicit pro-apoptotic effects have been a recent focus of investigation. PI3K/*AKT*/*mTOR* signaling pathway is highly activated in the retinal pigment epithelial cells of PVR [39]. The inhibitors of PI3K/*AKT*/*mTOR* signaling pathway, rapamycin and LY294002, could inhibited the PI3K/*AKT*/*mTOR* signaling pathway by reducing the levels of phosphorylation of *mTOR* pathway components [39]. Recent studies have implicated *AKT*/*mTOR* and *ERK*1/2 signaling pathways as mediators of rapamycin-dependent pro-apoptotic effects [40,41]. Our results suggested that the inhibition of the *AKT* or *ERK* signaling pathways could enhance the pro-apoptotic effect of rapamycin in HGF treated LECs cells. Rapamycin can strongly inhibit *AKT*/*mTOR* signaling pathway. We examined the change of *AKT*, *mTOR* and *ERK* phosphorylation caused by the administration of rapamycin after HGF stimulation and found that the levels of phosphorylated *AKT*, *mTOR*and *ERK* were remarkably decreased *in vitro*. To further investigate whether the pro-apoptosis is mediated by targeting *AKT*/*mTOR* and *ERK* pathway, we applied siRNA to knockdown the expression of *AKT* and *ERK* respectively and found it to make LECs more sensitive to rapamycin. Therefore, these data suggested that rapamycin may inhibit the *AKT*/*mTOR* and *ERK* pathways to induce pro-apoptotic effect.

### 2.4. JAK2/STAT3 Pathway Involvement in Apoptosis Promoted by Rapamycin in HGF-Treated LECs

**Figure 4:**
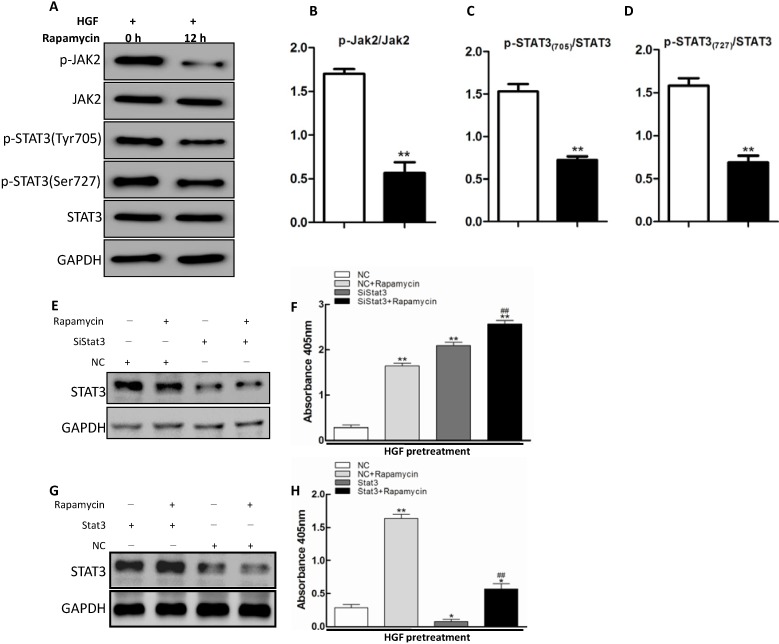
Rapamycin promoted apoptosis of HGF-treated LECs via *JAK2*/*STAT3* pathway. HGF (10 ng/mL)-pretreated LECs cells were stimulated with rapamycin (5 ng/mL) for 12 h or left untreated. (**A**) The levels of *JAK2*, *STAT3* and their phosphorylation status were detected by western blotting with GAPDH as a control; (**B**–**D**) Quantitative analysis of the p-*JAK2*/*JAK2*, p-*STAT3*_Tyr705_/*STAT3* and p-*STAT3*_Ser727_/*STAT3* ratios were shown in each lower panel (** *p* < 0.01, *t*-test); (**E**) Western Blotting, the expression of *STAT3* decreased significantly after *STAT3* siRNA transfection; (**F**) The LECs apoptosis rates were measured using Cell Death Detection ELISA PLUS kit as above indicating** *p* <0.01, compared with the NC cells. ^##^ *p* <0.01, comparedwith the LECs transfected with *STAT3*SiRNA. (*F =* 226.658, scheffe’s multiple range test); (**G**) *STAT3* forced transfection led to a significant increase in the *STAT3*expression; and (**H**) The LECs apoptosis rates were analyzed by Cell Death Detection ELISA PLUS kit * *p* < 0.05, ** *p* <0.01,compared with the NC(negative control) LECs. ^##^ *p* < 0.01, compared with the LECs in forced expression of *STAT3*(*F =* 144.99*,* scheffe’s multiple range test).

Subsequently, we investigated whether rapamycin inhibited the phosphorylation of *JAK2* and *STAT3* in HGF-treated LECs cells. Western blot analysis showed that rapamycin significantly inhibited the phosphorylation of *JAK2* and *STAT3* at both Tyr705 and Ser727 sites (Figure 4A). The fractions of p-*JAK2*/*JAK2*, p-*STAT3*_Tyr705_/*STAT3* as well as p-*STAT3*_Tyr727_/*STAT3* were all decreased (Figure 4B–D). To assess whether *STAT3* was involved in rapamycin-induced apoptosis of HGF-treated LECs cells, we applied siRNA to knockdown *STAT3* gene expression in LECs cells. As shown in Figure 4E, the western blot analysis indicated that transient transfection of *STAT3* siRNA into LECs cells resulted in a significant declination in the *STAT3* protein level respectively compared to the cells transfected with the control siRNA (Figure 4E). In addition, we also explore the function of rapamycin by forced expression of *STAT3* gene expression in LECs cells. As shown in Figure 4G, the western blot analysis indicated that transient transfection of *STAT3* into LECs cells resulted in a significant increase of the *STAT3* protein levels compared to the control group (Figure 4G), which was accompanied by a significant suppression of apoptosis in these cells (Figure 4H). Knockdown of *STAT3* significantly accelerated the apoptosis of LECs cells after HGF stimulation (Figure 4F), while forced expression of *STAT3* obviously inhibited apoptosis in HGF-treated LECs cells, indicating that *STAT3* played an important role in apoptosis of LECs cells after rapamycin treatment.

As a signal transducer and activator, *STAT3* regulates the expression of target genes involved in cell-cycle and apoptosis, and promotes cellular transformation as well as abnormal cell proliferation [42]. The JAK–STAT pathway is activated by cytokines and growth factors in human Lens Cells. AG-490, a specific inhibitor of *JAK2*/*STAT3*, suppressed growth factor–induced proliferation of LECs [43]. Combined treatments with rapamycin and *STAT3* gene silence significantly increases apoptosis in Bel-7402 Hepato-carcinoma cells, displaying more dramatic effect than any single treatment [38]. Our results suggested that *JAK2*/*STAT3* pathway was associated with apoptosis in HGF-pretreated LECs cells. The inhibition of *STAT3* could sensitize LECs cells to rapamycin treatment in the presence of HGF.

To date, there is no data on the effects of rapamycin on regulating *JAK2*/*STAT3* pathways in LEC apoptosis. For the first time, we found that rapamycin could significantly attenuate *JAK2* activation, and subsequently decrease the phosphorylation of *STAT3* in the presence of HGF *in vitro*. Further knockdown of *STAT3* with siRNA resulted in significantly activated cell apoptosis. All these findings suggest *JAK2*/*STAT3* pathway may play a crucial role in rapamycin-induced pro-apoptotic effects in HGF-treated LECs.

Nevertheless, in a recent study of myocardial ischemia-reperfusion injury [44], rapamycin appeared protection of the heart against previous studies [45,46]. They found rapamycin could promote the phosphorylation of *STAT3*, which is a central component of cardioprotection [47,48]. The reason for these opposing results is not clear. In our opinions, rapamycin may have different ways to trigger *JAK2*/*STAT3* pathway in different organs which is still unknown. Since rapamycin concentration and timing of its administration during ischemia-reperfusion injury may contribute opposing effects [49,50], further studies are needed to demonstrate whether there was contribution from another pathway besides *JAK2*/*STAT3* in rapamycin induced-apoptosis of HGF-treated LECs.

## 3. Experimental Section

### 3.1. Cell Culture, RNA Interference and Transient Transfection

The human LECs was kindly provided by YANG Chunbo (Tianjin medical university, eye institute). These cells were grown in Eagle’s minimum essential medium, containing 10% fetal bovine serum (FBS), 2 mM glutamine and 50 μg/mL gentamicin at 37 °C in a humidified 5% CO_2_ atmosphere, unless indicated. The cells were treated with HGF (10 ng/mL) for 12 h. Human HGF was obtained from Peprotech Inc. Silencer TM pre-designed siRNAs targeting human *AKT* (Cat. # 4390824), *ERK* (Cat. # 4390824) and *STAT3* (Cat. # 4390824) and negative control siRNA (Cat. # 4390843) were purchased from Ambion (Austin, TX, USA). LECs cells (2 × 10^5^ cells/well in 6-well plates) were transfected with siRNA of *AKT*, *ERK*1/2 and *STAT3* of which the final concentrations were 30 pM using Lipofectamine RNAiMAX Transfection Reagent (Cat. # 13778150) according to the manufacturer’s instructions, respectively. Protein samples were collected for Western blot analysis at 48 h after transfection.

### 3.2. Proliferation Assay

Cell proliferation was analyzed using a bromodeoxyuridine (BrdU) incorporation assay. LECs cells (1 × 10^4^ cells/well) pretreated with HGF (10 ng/mL) culturing in 96-tissue culture plates were untreated or treated with increasing amounts of rapamycin as indicated for different time points (24, 48 and 72 h), afterwards, the cells were starved in a Serum free medium (SFM), which was replaced 24 h later with a test medium consisting of SFM and the SFM was used as the negative control. The cells were incubated for 48 h before the addition of BrdU labeling solution and then were incubated for further 24 h. Then, the proliferation rate was measured (Cell Proliferation ELISA BrdUkit, Catalog No. 11647229001, Roche, Mannheim, Germany). Quadruplicates were used for each condition, and the experiments were repeated at least three times using different samples.

### 3.3. Cell Viability Assays

The change of LECs morphology and structure under different conditions was observed by H&E staining. A total of 2 × 10^4^ cells were seeded onto 9 × 9 mm coverslips. After 24 h of pretreatment with HGF, cells were untreated or treated with increasing amount of rapamycin as indicated for different cessions (24, 48 and 72 h). Afterwards, slides were immersed in 4% paraformaldehyde for 15 min, and then H&E staining was performed.

To check the viability, we used the LIVE & DEAD Viability/Cytotoxicity assay, which determines intracellular esterase activity and plasma membrane integrity. The cells were stained with thidium homodimer-1 and calcein AM according to kit instructions, after 30 min of incubation, they were imaged under fluorescence microscopy (Olympus BX51).

### 3.4. Apoptosis Assays

The percentage of apoptosis cells in each sample was determined with an Annexin-V-FLUOS Staining kit. (Roche, Catalog No. 11858777001, Mannheim, Germany) In brief, 1 × 10^6^ harvested cells were resuspended in 100 μL of the binding buffer, and then 2 μL Annexin V-FITC and 2 μL propidium iodide (PI, 20 mg/mL) were added. The tubes were incubated for 15 min at room temperature in the dark. Finally, binding buffer (400 μL) was added to each reaction tube and the cells were analyzed using a FACS Calibur Flow Cytometer with BD Cell Quest Pro Software (BD Biosciences, San Jose, CA, USA).

The terminal deoxyncleotidyl transferasemediated dUTP nick end labeling (TUNEL) assay was performed to confirm the existence of apoptotic cells after rapamycin simulated. 2 × 10^4^ cells were seeded onto coverslips. After 24 h of pretreatment with HGF, cells were untreated or treated with Rapamycin as indicated for different time points (24, 48 and 72 h). Apoptotic cells were visualized with green fluorescent under a fluorescence microscope. Nuclei were counterstained with DAPI (4',6-diamidino-2-phenylindole). The positive control of apoptosis was pretreated with DNase I to enzymatically induce DNA strand breaks.

Apoptosis was also quantified using the Cell Death Detection ELISA PLUS kit (Roche Applied Science, Catalog No. 11774425001, Mannheim, Germany) following the manufacturer’s protocol. This assay determines apoptosis by measuring mono- and oligonucleosomes in the lysates of apoptotic cells. The cell lysates were placed into a streptavidin-coated microplate and incubated with a mixture of anti-histone/biotin and anti-DNA/peroxidase. Absorbance was measured at 405 nm.

### 3.5. Western Blot Analysis

Cells were lysed in RIPA buffer (1% Nonidet P-40, 0.5% sodium deoxycholate, 0.1% SDS in PBS). A complete protease inhibitor mixture (Catalog No. 04693159001, Roche Applied Science, Mannheim, Germany) was added to lyse buffer before the analysis was carried out. Protein concentration was determined by the BCA protein assay (Catalog No. 23228, Pierce, Thermo Scientific, Rockford, IL, USA). Equal amounts of proteins were separated into 10% SDS-polyacrylamide gels, transferred to polyvinylidene fluoride membranes followed by incubation with: α-phospho-*STAT3*, α-*STAT3*, α-phospho *AKT*, *AKT*, α-phospho *ERK* ,*ERK* αphospho-*mTOR*, *mTOR* GAPDH(all from Cell Signaling Technology, Danvers, MA, USA), blots were developed using an ECL Plus kit (GE Healthcare, Freiburg, Germany). For quantification of protein intensities, WB images were captured using MultiSpectral Imaging System (EC3 410, UVP, Upland, CA, USA) and analyzed with the Lab Image 1D software (Kapelan Bio-Imaging Solutions, Leipzig, Germany).

### 3.6. Statistics

Statistical significance was determined by scheffe’s multiple range test and the two-tailed Student *t* test. All experiments were performed at least three times. *p* values of ≤0.05 were considered significant.

## 4. Conclusions

In summary, rapamycin could induce the apoptosis of human lens cells stimulated by HGF. It works by inhibiting the pathway of *AKT*/*mTOR* ,*ERK* and JAK/*STAT3* respectively.These findings would benefit its possible application in future to prevent PCO. 
